# Influence of Cognitive Functioning on Powered Mobility Device Use: Protocol for a Systematic Review

**DOI:** 10.2196/16534

**Published:** 2020-03-25

**Authors:** Alice Pellichero, Lisa K Kenyon, Krista Lynn Best, Éric Sorita, Marie-Eve Lamontagne, Marie Denise Lavoie, François Routhier

**Affiliations:** 1 Center for Interdisciplinary Research in Rehabilitation and Social Integration Québec, QC Canada; 2 Université Laval Quebec, QC Canada; 3 The Department of Physical Therapy Grand Valley State University Grand Rapids, MI United States; 4 Laboratoire EA 4136 Handicap Activité Cognition Santé Université de Bordeaux Bordeaux France; 5 Bibliothèque Université Laval Québec, QC Canada

**Keywords:** cognitive functioning, power mobility devices, clinicians, nurses

## Abstract

**Background:**

Power mobility devices (PMD) are critical to achieving independent mobility and social participation for many individuals who have trouble walking. Provision of PMDs is complex, with cognitive functioning expressed by clinicians as a major concern. Even if PMD use can be predicted by the level of cognitive functioning, outcome tools used to assess readiness do not consider how cognitive functioning may affect PMD use.

**Objective:**

The specific aims of this review are to identify existing assessments used to assess cognitive functioning and PMD use, classify cognitive functions that are identified within existing assessments related to PMD use, and explore the relationships between cognitive functioning (ie, executive functions and attention) and PMD use.

**Methods:**

A systematic review will be conducted using the electronic databases MEDLINE (Ovid), CINAHL, Embase, PsycINFO (Ovid), and Web of Science based on the concepts of PMD performance and capacity, and cognitive functioning. To be included, studies must have: a sample of PMD users (inclusive of age and diagnoses), an assessment of cognitive functioning, and an assessment of PMD capacity or performance. The International Classification of Functioning, Disability and Health will be used to classify cognitive functions. Study quality will be assessed using the Mixed Methods Appraisal Tool. Qualitative and quantitative studies will be analyzed in a complementary manner depending on their designs; a result-based convergent synthesis design will be applied.

**Results:**

This proposed systematic review protocol has been registered in PROSPERO (CRD42019118957). It was funded by the Quebec Rehabilitation Research Network and approved on February 2019.

**Conclusions:**

Results will inform the development of a PMD driving program that aims to enhance cognition. The results of this study will enhance understanding of the influence of cognitive functioning on PMD use and will support the clinical practice in choosing appropriate evaluative tools.

**Trial Registration:**

PROSPERO CRD42019118957; https://www.crd.york.ac.uk/PROSPERO/display_record.php? RecordID=118957

**International Registered Report Identifier (IRRID):**

DERR1-10.2196/16534

## Introduction

Individuals with mobility impairments can benefit from power mobility devices (PMD), such as powered wheelchairs; scooters [1]; and, specifically for children, adapted ride-on toys [2]. A national survey in Canada stated that, in 2016, approximately 160,000 individuals 15 years of age and older used a PMD (42,400 users of powered wheelchairs and 108,550 users of scooters) [3]. The prevalence of PMD use is expected to increase for both older adults, as the population continues to age [3], and children, as recommendations on early PMD provision are increasing [4].

According to the “Convention on the Rights of Persons with Disabilities” PMD use is critical for independent mobility [5]. In the International Classification of Functioning, Disability and Health (ICF) framework, mobility is described within the activities and participation chapter (d4). Mobility requiring use of a PMD is defined in “Moving around using equipment” (d465) as “moving the whole body from place to place, on any surface or space, by using specific devices designed to facilitate moving or create other ways of moving around, such as moving down the street in a wheelchair or a walker” [6]. The impact of PMD use is considerable, including the possibility to move throughout the user’s environment [7,8]. Optimal PMD use can also enhance mobility confidence and participation in various occupations [9-11]. Thus, using a PMD facilitates autonomy, independent living, and social participation in all aspects of life for children, adults, and older adults [5,12-14].

For example, the association between PMD use and participation has been demonstrated for older adults. Sund et al [11] conducted a prospective study and investigated the influence of PMDs over a period of 1 year. Among community-dwelling older adults, PMD use was associated with an increased frequency of grocery shopping and going for a walk, as well as other aspects of everyday life such as going to a restaurant, sending letters at the post office, going to the bank, and visiting family and friends became easier after PMD acquisition [11]. Additionally, Rossen et al [15] conducted a qualitative study exploring how PMD users experience their everyday life and how PMDs influence their daily occupations. The study reported that community-dwelling older adults reported a satisfaction with well-being, self-esteem, dignity, and social life that was associated with using a PMD [15].

However, to benefit from PMD use, a person must first obtain the device, which often requires a prescription from a health care professional and adaptations to their environment, and then demonstrate that they have the capacity to use it safely. For the purpose of this systematic review, PMD use encompasses capacity (ie, what a person can do in a standard environment) and performance (ie, what a person actually does in their actual environment) as defined by the ICF [16]. Accordingly, driving a PMD involves complex interactions between the person (social and cognitive factors), the environment, and the device itself. Therefore, PMD provision requires careful consideration of the diagnoses and prognoses; motor, cognitive, and perceptual capacities; and the built and social environments [17]. However, in practice, occupational therapists often report feelings of uncertainty when considering safety, autonomy, and risk through PMD acquisition [18].

Cognitive functioning is the major concern expressed by clinicians who prescribe PMDs [18,19], as learning new skills (ie, capacity) and applying the skills in the real world (ie, performance) requires adequate cognitive abilities. There is evidence to suggest that successful PMD use can be predicted by the level of cognitive functioning [20]. For example, Cullen et al [20] demonstrated that cognitive functions such as memory and visual perception upon PMD provision predicted frequency of PMD use 1 month later. However, one evaluation of cognition was based on an index score that combines multiple tools. Through evaluation of a PMD training program among individuals living in long-term care, Mendoza et al [21] also reported an increased number of accidents among PMD users who had executive dysfunction. Despite the perceived importance of cognition, global or specific cognitive functions required for PMD use remain unclear.

Clear clinical guidelines related to cognition and PMDs are limited by a dearth of literature that has not yet been adequately synthesized. Furthermore, existing PMD use assessment tools focus predominantly on motor skills and performance-based outcomes, and seldom consider how cognitive functions and application of knowledge (ie, executive functioning, problem solving) could influence PMD driving. For example, the Power Mobility Indoor Driving Assessment [22] and the Power Mobility Community Driving Assessment considers whether more training is required; the Wheelchair Skills Test assesses specific driving skills [23]; the Power Mobility Road Test evaluates driving capacities in structured and unstructured environments [24]; and the Wheelchair Use Confidence scale measures self-efficacy for using a PMD [25]. In addition, the Functional Evaluation Rating Scale evaluates driving performance in simulated programs [26]. However, existing assessment tools do not consider how cognitive functioning may affect PMD use [27]. Consequently, subjective clinical judgment often plays a central role in determining if an individual has the necessary cognitive functions for using a PMD [18].

Given that cognitive functioning is fundamental to using a PMD and that decision making around PMD provision often relies on clinical judgement, it is critical to gain a better understanding of the relationships between cognitive functioning and PMD use, which is important for the development of assessment tools and training programs. To our knowledge, there has not been a systematic review describing the relationship between PMD use and cognitive functioning.

The specific aims of this review are to identify existing assessments used to evaluate cognitive functioning and PMD use; classify, according to the ICF, cognitive functions that are identified within existing assessments related to PMD use; and explore the relationships between cognitive functioning (ie, executive function, attention) and PMD use.

## Methods

### Prospero Registration and PRISMA-P Statement

The present protocol has been registered within the PROSPERO database (CRD42019118957). Given that there are no guidelines for mixed-method reviews [28], this review will follow the relevant domains of the PRISMA-P (Preferred Reporting Items for Systematic Reviews and Meta-Analyses Protocols) statement for quantitative aspects [29] (Multimedia Appendix 1), and the relevant domains of the Enhancing Transparency in Reporting the Synthesis of Qualitative Research (ENTREQ) statement for qualitative aspects [30].

### Literature Search

A librarian contributed to the development of the search strategy. Appropriate keywords were selected according to Medical Subject Headings (MeSH) terms, terms used in existing studies on cognition and PMD use, and the “Mental functions” and “Learning and applying knowledge” chapters of the ICF. The search was conducted in online databases including MEDLINE (Ovid), CINAHL, Embase, PsycINFO (Ovid), Scopus, and Web of Science. The search strategy included the concepts: “PMD”, “cognitive functioning”, and related synonyms. In each database, the subject headings related to the two concepts were used. The keywords and writing rules (eg, truncation, quotation marks, operators) were adapted for each database. An example of the search strategy is provided in Multimedia Appendix 2. The results were searched independently by two authors (AP and MDL) to identify relevant studies. All searches were documented including terms used and the number of hits or studies obtained.

### Eligibility Criteria

The PICOS (Population, Intervention, Comparison, Outcomes, and Study Designs) structured approach [29] was used to frame inclusion and exclusion criteria. Included studies must: be scientific peer-reviewed manuscripts, dissertations, or theses (including all quantitative and qualitative methods and study designs); present original data; be written in English or French; include a sample of PMD users (inclusive of age and diagnoses); assess cognitive functioning; and assess PMD capacity or performance. No restriction will be applied regarding year of publication. Studies not involving human subjects will be excluded.

### Data Management

The data will be imported from the databases in Endnote reference management software (version X9). Then references will be exported to Covidence systematic review software (Veritas Health Innovation, Melbourne, Australia), where duplicates will be removed automatically based on the title of the references. Remaining duplicates will be deleted during the abstract and title screening.

### Screening and Selection Process

Titles and abstracts will be screened for eligibility by two independent reviewers (AP and LK). The full text of all relevant studies will be retrieved and independently assessed for inclusion by two reviewers (AP and LK). Any disagreement in study eligibility will be resolved through discussion with a third reviewer (KB). References of all considered studies will be hand searched to identify any relevant reports missed in the search strategy.

### Data Extraction

Data will be extracted independently by two reviewers (AP and LK) into study-specific extraction tables. The same data extraction approach will be applied across all studies following a standard data extraction template, but with flexibility according to various methodologies and designs [28]. Study designs will be extracted according to Portney and Watkins’ definitions [31]. All tables will include the following general categories: author; year of publication; country; study design; purpose of study; type of power mobility device (wheelchair or scooter); participant demographics (sample size, sex, age, marital status, diagnosis); primary outcome: cognitive functions (classified using the ICF and including outcome tools when applicable) (Multimedia Appendix 3); and secondary outcome: PMD use (including outcome tools when applicable). Specific tables for randomized control design, pre-post design, and intervention design will include categories such as intervention, control group, and outcome measure. Only select qualitative data will be extracted according to the specific aims of the protocol (ie, cognition and power wheelchair mobility outcomes) [32]. For example, phrase and keywords (codes) related to cognition and PMD capacity and performance will be reviewed and extracted [33]. The authors will read each article repeatedly to ensure that all concepts and relationships are explored [34]. Discrepancies will be identified and resolved through discussion, using a mediator (KB) when necessary. Missing data will be requested from study authors.

### Data Analysis

Embracing no restriction related to the study purpose and design assumes complementarity between methodologies, as such a transparent and systematic process will be used [28,35]. The studies will be analyzed in the same time and in a complementary manner depending on their design. Then the results of both syntheses will be integrated during a final synthesis. A result-based convergent synthesis design will be applied [36]. This design implies that qualitative and quantitative studies are analyzed separately using different synthesis methods, and that the results of the qualitative synthesis informed the quantitative synthesis [37]. First, qualitative and quantitative data will be analyzed separately. Codes extracted from all qualitative studies will be organized accounting for similarities and differences in the study findings and will lead to new interpretations of the phenomena studied [30]. For quantitative data, if studies are sufficiently homogenous, quantitative synthesis will be used (aggregate level data) and correlations between cognitive functioning and PMD measures will be calculated with SPSS Statistics (SPSS Inc, Chicago, Illinois). Second, a narrative synthesis will be integrated to merge the results of both qualitative and quantitative syntheses, which will then be combined using a third synthesis in a convergent manner [36]. Throughout analyses multiple researchers will be involved in peer debriefings. Consensus will be reached among three researches (AP, LK, and KB) to assure reliability and trustworthiness. The interpretation of the results will occur in the discussion section.

### Critical Appraisal

The studies will be organized by study design in descending order from the highest level of evidence to the lowest level according to an evidence-based practice toolkit [38]. The methodological quality of each included study will be appraised using the Mixed Methods Appraisal Tool (MMAT), evaluating qualitative and quantitative designs [39]. It is noteworthy that this tool is currently being updated; if the new version is available during this study, the most recent version will be used. Methodological limitations identified in primary studies will be taken into account to discuss the results and conclusions regarding the relationship between cognitive functions and PMD use. This systematic review does not have restrictions related to the study designs; therefore, the methodological quality of each article will be essential to the interpretation of the results. The two appraisals will be completed independently by two authors (AP and LK). Discrepancies will be identified and resolved through discussion with a third author (KB) when necessary.

### Ethical Considerations

There are no ethical issues of concern in this secondary analysis of published evidence.

## Results

The review has been designed according to the Cochrane method [40], such that each step will be performed in duplicates (screening and selection, data extraction and data analysis). Transparency will be enhanced by regular team meetings and presentations on emerging findings at internal seminars, as well as by sharing the findings with an advisory group. All steps and decisions such as discussions about keywords or the exclusion of a study will be recorded in a logbook. None of the authors have conflicts of interest that would affect their interpretation of evidence.

## Discussion

This paper describes the protocol for a systematic review aiming to identify the cognitive functions that are currently assessed before PMD provision and to explore the relationships between cognitive functions and PMD use. Results of this study will improve knowledge about the assessment of cognitive functioning and the relationship between cognitive functions and PMD use. It is apparent that cognitive functioning is required for PMD use. For example, Bottos et al [41] assessed the effects of early provision of a powered wheelchair, and found that children classified as “normal” or “mild learning disability” according to their IQ achieved independent use of PMD easily and rapidly. However, there is a need to better understand the influence of specific cognitive functions on PMD use, and to determine cognitive functions that predict successful PMD use. There is little evidence describing an explicit relationship between cognitive functioning and PMD use. Moreover, the most commonly used assessments of readiness for PMD use [27] focus on PMD capacity or performance outcomes (ie, assess *activity and participation* of a wheelchair user) [19], and may overlook modifiable cognitive functions.

Existing assessments of PMD use and assessments of cognitive functioning identified in this systematic review will be classified according to the ICF. Therefore, the results of this systematic review may guide therapists in the selection of outcome tools for PMD screening and assessment. Moreover, identification of important cognitive functions may provide valuable insight into the development of new PMD driving interventions, and specific cognitive functions (eg, problem solving and executive functioning) may be directly targeted using safe and specialized approaches such as wheelchair simulator environments and virtual reality.

Realization of the proposed systematic review is the first iterative step within a larger program of research that will lead to the development of a PMD training program that targets cognitive functioning. The Medical Research Council methodological framework will guide the development and the evaluation of a novel PMD training program that considers important cognitive functions [42]. This framework follows four phases for the development of complex interventions including: the theoretical and developmental phase (phase I), the feasibility phase (phase II), the evaluation phase (phase III), and the long-term implementation phase (phase IV) [43]. The theoretical phase suggests conducting a systematic review to synthesize existing knowledge. Findings from this review will be used to design the prototype for a new PMD training program that targets cognitive functioning, which will then be refined and evaluated with key stakeholders and experts (eg, PMD users, caregivers, clinicians) through focus groups and Delphi surveys.

The results from this systematic review will enhance the understanding of the influence of cognitive functions on PMD use, which is critical for the development of assessment tools and training programs. Results of this systematic review may inform the development of clinical practice guidelines and training programs that consider cognition and the development of smart wheelchairs. There may be practical applications for PMD users, caregivers, and clinicians.

One limitation of this review is that the strategy will not include literature. Moreover, the broad population being targeted (ie, individuals with cognitive and physical impairments) may pose some challenges. However, we chose to include participants of all ages and diagnoses to not restrict or exclude relevant studies. If data are available, subgroup analyses and descriptions will be considered to describe specific relationships between cognitive functioning and PMD use in different populations (ie, age, sex, diagnoses). The anticipated high variability between existing assessments such as qualitative descriptions versus quantitative outcome tools may also limit the ability to make comparisons between studies and to synthesize findings. Finally, inclusion of qualitative and quantitative studies will potentially limit our ability to make any conclusions regarding strength and magnitude of relationship between cognitive functions and PMD use.

This systematic review will aim to explore the relationships between cognitive functioning and PMD use. The results of this study will improve knowledge about the influence of cognitive functions on PMD use. This protocol provides a detailed description of the methods that will be used to conduct the systematic review thus ensuring transparency and a priori directions for future research.

## Appendix

Multimedia Appendix 1 Prisma P Checklist 2015.

Multimedia Appendix 2 Example of search strategy in MEDLINE/Ovid database.

Multimedia Appendix 3 Classification of cognitive functions (mental functions) according to different levels of the International classification of functioning.

Multimedia Appendix 4 Previous peer-review report from PROSPERO.

## Abbreviations

ENTREQ: Enhancing Transparency in Reporting the Synthesis of Qualitative Research
ICF: International Classification of Functioning, Disability and Health
MeSH: Medical Subject Headings
PICOS: Population, Intervention, Comparison, Outcomes, and Study Designs
PMD: power mobility device
PRISMA-P: Preferred Reporting Items for Systematic Reviews and Meta-Analyses Protocols.
