# Supplementary material for: Influence of Cognitive Functioning on Powered Mobility Device Use: Protocol for a Systematic Review
Source: JMIR Res Protoc. 2020 Mar 25;9(3):e16534. doi: 10.2196/16534 (PMC7142732; doi:10.2196/16534)
Supplement: Multimedia Appendix 2 [file resprot_v9i3e16534_app2.docx]

**APPENDIX I - Table 1** – Example of search strategy in MEDLINE/Ovid database.

**medline (ovid)**

|  | "electric wheelchair" or "motorized wheelchair" or "power* mobility" or "power* wheelchair" or scooter* | [ti. ab.] |
| --- | --- | --- |
|  | Wheelchairs/ | MESH |
|  | **1 or 2** |  |
|  | "ability to learn*" or anticipat* or attention* or automatism* or aware* or cognition* or ((abilit* or control* or defect* or deficit* or disabilit* or disorder* or dysfunction* or function* or impair* or performance* or process* or rehabilitation or skill*) adj5 cognitive) or concentrat* or conditioning or "decision making" or "decision-making" **or (**(depth or distance or space) adj5 perception*) or "discrimination learn*" or ((function* or syndrome) adj5 dysexecutive) or emotion* or ((control* or dysfunction* or disorder* or function*) adj5 executive) or expectation* or experienc* or "intellectual function*" or memory or "mental concentration" or "mental function*" or orientation or perception* or ((disorder* or function* or orientation) adj5 perceptual) or "problem solving" or "problem-solving" or ((discrimination or learn* or memory or navigation* or orientation* or perception*) adj5 spatial) | [ti. ab.] |
|  | anticipation, psychological/ | MESH |
|  | attention/ | MESH |
|  | automatism/ | MESH |
|  | awareness/ | MESH |
|  | cognition disorders/ | MESH |
|  | cognition/ | MESH |
|  | cognitive dysfunction/ | MESH |
|  | "conditioning (psychology)"/ | MESH |
|  | decision making/ | MESH |
|  | depth perception/ | MESH |
|  | discrimination learning/ | MESH |
|  | distance perception/ | MESH |
|  | emotions/ | MESH |
|  | executive function/ | MESH |
|  | Intelligence/ | MESH |
|  | memory/ | MESH |
|  | mental processes/ | MESH |
|  | orientation/ | MESH |
|  | orientation, spatial/ | MESH |
|  | perception/ | MESH |
|  | problem solving/ | MESH |
|  | space perception/ | MESH |
|  | spatial learning/ | MESH |
|  | spatial memory/ | MESH |
|  | spatial navigation/ | MESH |
|  | 5/29 or | MESH |
|  | 4 or 30 |  |
|  | 3 AND 31 |  |
