# Supplementary material for: Influence of Cognitive Functioning on Powered Mobility Device Use: Protocol for a Systematic Review
Source: JMIR Res Protoc. 2020 Mar 25;9(3):e16534. doi: 10.2196/16534 (PMC7142732; doi:10.2196/16534)
Supplement: Multimedia Appendix 3 [file resprot_v9i3e16534_app3.docx]

**APPENDIX III -** Classification of cognitive functions (mental functions) according to different levels of the ICF

| Mental functions | Global mental functions (b110-b139) | b110 consciousness functions | b1100 state of consciousness |
| --- | --- | --- | --- |
|  |  |  | b1101 continuity of consciousness |
|  |  |  | b1102 quality of consciousness |
|  |  |  | b1108 other specifies |
|  |  |  | b1109 unspecified |
|  |  | b114 orientation functions | b1140 orientation to time |
|  |  |  | b1141 orientation to place |
|  |  |  | b1142 orientation to person |
|  |  |  | b1148 other specifies |
|  |  |  | b1149 unspecified |
|  |  | b117 intellectual functions |  |
|  |  | b122 global psychosocial functions |  |
|  |  | b126 temperament and personality functions | b1260 extraversion |
|  |  |  | b1261 agreeableness |
|  |  |  | b1262 consciousness |
|  |  |  | b1263 psychic stability |
|  |  |  | b1264 openness to experience |
|  |  |  | b1265 optimism |
|  |  |  | b1266 confidence |
|  |  |  | b1267 trustworthiness |
|  |  |  | b1268 other specifies |
|  |  |  | b1269 unspecified |
|  |  | b130 energy and drive functions | b1300 energy level |
|  |  |  | b1301 motivation |
|  |  |  | b1302 appetite |
|  |  |  | b1303 craving |
|  |  |  | b1304 impulse control |
|  |  |  | b1308 other specifies |
|  |  |  | b1309 unspecified |
|  |  | b134 sleep functions | b1340 amount of sleep |
|  |  |  | b1341 onset of sleep |
|  |  |  | b1342 maintenance of sleep |
|  |  |  | b1343 quality of sleep |
|  |  |  | b1344 functions involving the sleep cycle |
|  |  |  | b1348 other specifies |
|  |  |  | b1349 unspecified |
|  |  | b139 global functions, other specified and unspecified | b1400 sustaining attention |
|  | Specific mental functions (b140-b189) | b140 attention functions | b1401 shifting attention |
|  |  |  | b1402 dividing attention |
|  |  |  | b1403 sharing attention |
|  |  |  | b1408 other specifies |
|  |  |  | b1409 unspecified |
|  |  | b144 memory functions | b1440 short-term memory |
|  |  |  | b1441 long-term memory |
|  |  |  | b1442 retrieval memory |
|  |  |  | b1448 other specifies |
|  |  |  | b1449 unspecified |
|  |  | b147 psychomotor functions | b1470 psychomotor control |
|  |  |  | b1471 quality of psychomotor functions |
|  |  |  | b1478 other specifies |
|  |  |  | b1479 unspecified |
|  |  | b152 emotional functions | b1520 appropriateness of emotion |
|  |  |  | b1521 regulation of emotion |
|  |  |  | b1522 range of emotion |
|  |  |  | b1528 other specifies |
|  |  |  | b1529 unspecified |
|  |  | b156 perceptual functions | b1560 auditory perception |
|  |  |  | b1561 visual perception |
|  |  |  | b1562 olfactory perception |
|  |  |  | b1563 gustatory perception |
|  |  |  | b1564 tactile perception |
|  |  |  | b1565 visuospatial perception |
|  |  |  | b1568 other specifies |
|  |  |  | b1569 unspecified |
|  |  | b160 thought functions | b1600 pace of thought |
|  |  |  | b1601 form of thought |
|  |  |  | b1602 content of thought |
|  |  |  | b1603 control of thought |
|  |  |  | b1608 other specifies |
|  |  |  | b1609 unspecified |
|  |  | b164 higher-level cognitive functions | b1640 abstraction |
|  |  |  | b1641 organization and planning |
|  |  |  | b1642 time management |
|  |  |  | b1643 cognitive flexibility |
|  |  |  | b1644 insight |
|  |  |  | b1645 judgement |
|  |  |  | b1646 problem-solving |
|  |  |  | b1648 other specifies |
|  |  |  | b1649 unspecified |
|  |  | b167 mental functions of language | b1670 reception of language |
|  |  |  | b1671 expression of language |
|  |  |  | b1672 integrative language functions |
|  |  |  | b1678 other specifies |
|  |  |  | b1679 unspecified |
|  |  | b172 calculation functions | b1720 simple calculation |
|  |  |  | b1721 complex calculation |
|  |  |  | b1728 other specifies |
|  |  |  | b1729 unspecified |
|  |  | b176 mental functions of sequencing complex movements |  |
|  |  | b180 experience of self and time functions | b1800 experience of self |
|  |  |  | b1801 body image |
|  |  |  | b1802 experience of time |
|  |  |  | b1808 other specifies |
|  |  |  | b1809 unspecified |
|  |  | b189 specific mental functions, other specified and unspecified |  |
|  |  | b198 mental functions other specified |  |
|  |  | b199 mental functions unspecified |  |
