# Supplementary material for: Influence of Cognitive Functioning on Powered Mobility Device Use: Protocol for a Systematic Review
Source: JMIR Res Protoc. 2020 Mar 25;9(3):e16534. doi: 10.2196/16534 (PMC7142732; doi:10.2196/16534)
Supplement: Multimedia Appendix 4 [file resprot_v9i3e16534_app4.pdf]

PROSPERO
International prospective register of systematic reviews

Dear Dr Best,

Thank you for submitting details of your systematic review "Influence of cognitive functions on powered mobility device use: a systematic review" to the PROSPERO register. To assist us in processing your application, please could you clarify the following:

Please review Field #16 (Searches) and complete it in accordance with PROSPERO guidance. In particular you should provide details of any restrictions or limitations that were imposed on your searches, especially publication dates and languages. Similarly, if no limits were used, please state this clearly. It would also be good (but not essential) if you could provide a summary of your search terms.

Please could you also complete field #28 to say exactly what you are doing, rather than a generic statement. Enough information should be provided to indicate that a formal synthesis is planned (whether that be quantitative, qualitative or formal narrative synthesis). You should include details for your specific review.

Strategy for data synthesis: field #28
Give the planned general approach to synthesis, e.g. whether aggregate or individual participant data will be used and whether a quantitative or narrative (descriptive) synthesis is planned. Mention software, tools and statistical tests you plan to use. Include the number of researchers involved and how discrepancies will be resolved.

You need to be more clear in the type of synthesis you wish to perform, as the current information is a bit vague. You have also ticked in field #30 to state that a meta-analysis will be conducted, yet there is no mention of this in field #28 - please clarify whether or not this will be produced, and if so, how?

The record has been referred to you. Please could you login to your

PROSPERO account, make the appropriate amendments (include an explanation in the revision note) and SUBMIT it for assessment.

Yours sincerely,

PROSPERO Administrator
Centre for Reviews and Dissemination
University of York
York YO10 5DD
t: +44 (0) 1904 321049
e: CRD-register@york.ac.uk
www.york.ac.uk/inst/crd

PROSPERO is funded by the National Institute for Health Research and produced by CRD, an academic department of the University of York.

Email disclaimer: https://www.york.ac.uk/docs/disclaimer/email.htm

---

PROSPERO
This information has been provided by the named contact for this review. CRD has accepted this information in good faith and registered the review in PROSPERO. CRD bears no responsibility or liability for the content of this registration record, any associated files or external websites.
